# A severe dismantling of LGBTQI+ health equity and equality: impact of new U.S. policies on the global response to HIV

**DOI:** 10.1002/jia2.26485

**Published:** 2025-05-15

**Authors:** Sean Robert Cahill

**Affiliations:** ^1^ Health Policy Research The Fenway Institute Boston Massachusetts USA; ^2^ Health Law Policy and Management Boston University School of Public Health Boston Massachusetts USA; ^3^ Bouve College of Health Sciences Northeastern University Boston Massachusetts USA

1

Since 20 January 2025, the new U.S. administration has deployed what is likely to be an illegal and unconstitutional “shock and awe” strategy to effectively dismantle entire government agencies that fund healthcare and humanitarian aid around the world, and to repeal policies and programmes supporting LGBTQI+ health equity and equality both in domestic and global policies. Although several executive orders target the so‐called promotion of “gender ideology,” and transgender people in particular, U.S. agencies and private entities are overcomplying, even in the absence of rules and regulations requiring them to do so, ending programmes supporting LGBQTI+ individuals. This includes research on health disparities affecting LGBTQI+ people in the United States and around the world. Global programmes supporting LGBTQI+ communities, a key foreign policy goal of previous U.S. administrations, have been abruptly ended, leaving sexual and gender minority people even more vulnerable than they already were.

Specifically, on his first day in office, President Trump issued three executive orders that affect health equity in the United States. The first opposed alleged “gender ideology extremism” [1]. It defined sex as referring to “an individual's immutable biological classification as exclusively male and female,” and stated that “[a]gencies shall take all necessary steps, as permitted by law, to end the Federal funding of gender ideology.” This has led to the defunding of hundreds of existing U.S. National Institutes of Health (NIH) research grants that included LGBTQI+ participants and HIV prevention and care research studies, and to new applications being abruptly removed from NIH consideration. It could also eventually restrict healthcare entities’ ability to provide gender‐affirming care.

Executive orders related to “gender ideology extremism” [2] repealed a January 2021 executive order that prohibited discrimination by the federal government on the basis of sexual orientation and gender identity (SOGI) [3]. The rescinding of SOGI non‐discrimination under U.S. law could have a grave impact on the ability of LGBTQI+ people to find employment, earn an income, and access housing, healthcare and other public services. While the 2020 U.S. Supreme Court ruling in *Bostock v. Clayton County, Georgia*, prohibits anti‐gay and anti‐transgender discrimination in employment nationwide [4], LGBTQI+ Americans continue to experience routine discrimination in many settings. A 2022 survey found that more than one‐third of LGBTQI+ Americans reported experiencing discrimination in the past year. More than one in five respondents said that they delayed or avoided medical care due to experiences of discrimination [5].

A third executive order targeted Diversity, Equity and Inclusion (DEI) initiatives [6]. As a result of this EO, U.S. government agencies have removed information about racial and ethnic disparities from websites, and ordered the termination of many research studies examining such disparities, including HIV prevention research. While it will take months or even years to create and finalize rules and regulations to implement all of these executive orders, in response to them a number of government agencies and private entities have taken steps to roll back LGBTQI+ and racial equity health initiatives and access to care. For example, the U.S. Centers for Disease Control and Prevention (CDC) removed information related to LGBTQI+ health and racial and ethnic disparities from its website, including peer‐reviewed academic journal articles on these topics [7]. This action violates a number of U.S. laws, including the Public Health Service Act, which requires the U.S. Department of Health and Human Services, of which CDC is a part, to “assemble accurate data to assess research priorities,” including “data on study populations of clinical research, funded by or conducted at each national research institute and national center” (*Id*. § 282(b)(4)(B)). In addition to the direct impact of these new U.S. policies, a number of private healthcare institutions have stopped providing gender‐affirming care for youth, even though regulations to enforce the anti‐transgender executive orders have not yet been finalized [8]. Perhaps most devastating for LGBTQI+ people around the world has been the near‐total dismantling of the U.S. Agency for International Development (USAID). As of 7 March 2025, nearly all of USAID's staff and contractors had been fired, and only one‐tenth of its contracts were still in place [9].

Following its reauthorization in 2008, PEPFAR started supporting HIV prevention programmes for men who have sex with men (MSM) and transgender women, both populations at elevated risk for HIV acquisition. It also started collecting data on these populations to understand their burden of HIV in Africa and elsewhere. Then Senator Joe Biden was Senate Foreign Relations Committee Chair in 2008 when language calling on PEPFAR to address MSM was included in the reauthorization [10]. Under the Obama and Biden Administrations, promoting equal rights for LGBTQI+ people was a key goal of U.S. foreign policy. The Trump Administration has discontinued this approach and stopped funding LGBTQI+ organizations in other countries. Just days into his second term, President Trump ended all aid flowing through USAID. Secretary of State Marco Rubio then announced an exception for live‐saving medical care such as HIV treatment, but not for groups that promote DEI or focus on LGBTQI+ people. As a result, about 97% of some 127 Ugandan organizations that conduct HIV prevention and care work with LGBTQI+ communities lost all of their USAID funding, and most have had to close operations [11]. This will have devastating effects on HIV prevention and care in Uganda and around the world, where gay and bisexual men and transgender women are disproportionately burdened by HIV and are subject to violence and persecution by other members of society and by governmental actors.

In January, the President's Emergency Plan for AIDS Relief (PEPFAR), a programme that worked closely with USAID and CDC to prevent HIV and treat people living with HIV around the world, was put under a 90‐day review by administration officials, and its fate is unknown. PEPFAR had a $6.5 billion budget in fiscal year 2024. Some $4.8 billion of this was “bilateral aid” that went directly to 55 countries; 60% flowed through USAID, and the rest went to the CDC and the U.S. Department of Defense. The remaining $1.7 billion went to the Global Fund to Fight HIV, AIDS and Malaria [12]. While most of the PEPFAR funding through USAID was stopped, the fate of the other funding is unclear. A recent analysis estimates that ending PEPFAR funding in South Africa alone, where PEPFAR funding constitutes 18% of the country's HIV budget, “would cause an additional 601,000 HIV‐related deaths and 565,000 new HIV infections…over 10 years” [13].

While the current U.S. administration's regressive policies target “gender ideology” and DEI, they are having a much broader impact that is silencing and erasing research on health and disparities among LGBTQI+ people and among racial and ethnic minorities. The mass defunding of LGBTQI+‐related programming supported by USAID and other U.S. government programmes, massive layoffs and funding cuts at CDC's Division of HIV Prevention and USAID, and the promotion of homophobic and transphobic policies will exacerbate HIV epidemics globally.

## COMPETING INTERESTS

There are no competing interests.

## AUTHORS’ CONTRIBUTIONS

SRC conceptualized and wrote this viewpoint article.

## FUNDING

No funding supported the research or writing of this viewpoint article.

## DISCLAIMER

The views in this viewpoint article are those of the author alone and do not represent the views of The Fenway Institute, Boston University, Northeastern University or any of the other institutions with which Sean Robert Cahill is affiliated.

## Data Availability

Data sharing is not applicable to this article as no new data were created or analysed in this study.
